# Applications and Outcomes of Telehealth and Integrated Care in Men’s Health Urology

**DOI:** 10.2196/69095

**Published:** 2025-06-18

**Authors:** Bryce Bisset, Tarun Shriram, Meenakshi Davuluri, Hassan Azar, Jonathan Beilan, Reza Amin, Justin Houman

**Affiliations:** 1 Bastion Health, 400 Farmington Ave, Farmington, CT, 06032, United States, 1 2034489151; 2 College of Liberal Arts and Sciences, University of Connecticut, Storrs, CT, United States; 3 Department of Urology, Weill Cornell Medicine, New York, NY, United States; 4 Health Growth Advisory Network, Detroit, MI, United States; 5 Advanced Urology Institute, Largo, FL, United States; 6 Tower Urology, Cedars-Sinai Medical Center, 8635 W 3rd St, Suite #1W, Los Angeles, CA, 90048, United States, 1 7149287950

**Keywords:** telehealth, integrated care, men's health urology, trends in urology, quadruple aim

## Abstract

Men’s health, particularly in the domain of urology, faces significant challenges in access to care, patient outcomes, and cost efficiency. Despite advances in medical treatment, conditions such as prostate cancer remain a leading cause of cancer-related death among men, with African American men disproportionately affected at twice the mortality rate of other groups. Compounding these challenges is a critical shortage of urologists, with 62% of US counties lacking a practicing urologist and only 1 new urologist entering the field for every 10 retiring. This shortage results in delayed diagnoses, increased rates of advanced-stage conditions, and significant health disparities. To address these pressing issues, telehealth and technology-based integrated care models present a promising solution. Telehealth expands access to specialized urological care by overcoming geographical barriers and offering virtual consultations, at-home diagnostics, and continuous patient engagement. Artificial intelligence–driven tools further enhance the efficiency and accuracy of care delivery, improving provider experience by automating administrative tasks and facilitating early intervention through predictive analytics. Furthermore, remote patient monitoring devices provide accurate, cost-effective, and highly accessible alternatives. These innovations reduce provider burnout, lower health care costs, and, critically, improve patient outcomes. This paper explores the potential of telehealth and integrated care in men’s health urology as a practical pathway to bridging access gaps, enhancing care quality, and achieving cost savings. By leveraging digital health solutions, health care systems and employers can promote health equity, increase engagement, and ensure that all men receive timely and effective urological care.

## Introduction

Telehealth and integrated care are becoming increasingly essential to all areas of health care. Studies in men’s health and urology reveal that telehealth and integrated care are vital, especially as the prevalence of conditions such as benign prostatic hyperplasia, prostate cancer, and erectile dysfunction increases [1]. Telehealth, which involves delivering medical services remotely via telecommunications technology, enables remote patient-clinician interactions, monitoring, and management [2]. Integrated care is a coordinated approach where different health care providers and services are working together to deliver holistic, patient-centered care [3]. This model ensures that patients receive comprehensive care, addressing their immediate clinical needs and long-term health and wellness.

The adoption of telehealth and integrated care in urology may address several critical challenges: low interest in health engagement by men, a shortage of urologists, and limited access to care. Over 85 million men in the United States voiced concerns about cancer, reproductive, and sexual health, yet 55% do not make the effort to engage in preventive health care [4]. In addition, 29.8% of the urology workforce is 65 years and older, foreshadowing a massive shortage of urologists insufficient to meet the increasing demands of an aging population [5]. Fragmentation in health care limits access by causing inefficiencies and gaps in treatment due to uncoordinated care [6,7]. Telehealth and integrated care improve access to urologic care while enhancing the efficiency and quality of health care delivery. Patients can receive care at their time and place of choosing, provide at-home, confidential testing, and be more open to this experience due to ease of scheduling [8]. These approaches align with the Quadruple Aim in health care, which focuses on improving population health outcomes, enhancing patient care and experience, increasing provider satisfaction, and reducing health care costs [9].

Despite challenges such as some patient’s lack of high-speed internet, lack of sufficient video conferencing tools, lack of technology literacy, and the providers’ resistance to workflow changes, the evolving landscape of men’s health care necessitates the adoption of telehealth and integrated care in urology [10,11]. This paper highlights the transformative impact of telehealth and integrated care on urological care, particularly in addressing the Quadruple Aim in health care. We examine the historical and recent changes in health care delivery models, examine the benefits of these approaches in improving population health care access, and explore their effects on improved patient and provider experiences. In addition, we address the economic implications, focusing on how these models can reduce health care costs among patients and providers alike. We also identify the barriers to implementing telehealth and integrated care and propose potential solutions.

## Evolution of Telehealth and Integrated Care in Urology

**Figure 1.**
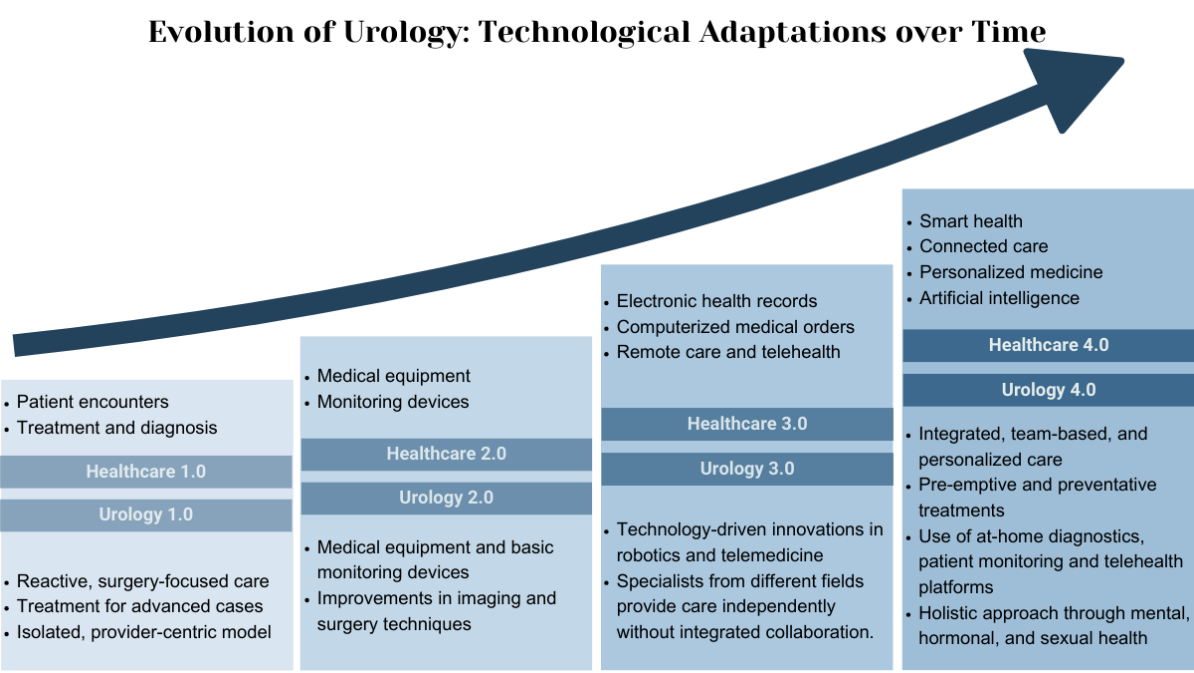
Major innovations in the development of health care and urology.

The evolution of care in urology is marked by significant advancements in medical technology and health care delivery models, similar to the evolution of health care. Initially, urological care heavily relied on in-person diagnostic visits, presenting challenges for patients with limited mobility or residing in remote areas. Figure 1 shows how Urology 1.0 defines this stage, identifiable by a lack of technology and reactive, provider-centered care. As medical equipment advanced, along with the invention of the telephone in the late 19th century, specialization and remote patient access allowed for a multidisciplinary approach to patient care in Urology 2.0. The mid-twentieth century included significant advancements in electronic communication that paved the way for teleradiology and video consultations, marking the beginning of modern telehealth applications in Urology 3.0 [12]. Accompanied by innovations in medical technology such as electronic health records, urologic care became more efficient and specialized for men’s health issues. Recent developments in integrated care models, medical devices, and artificial intelligence (AI)–powered smart health care enabled the personalized, holistic approach to urologic care defined by Urology 4.0 [13]. By 2012, millions of Americans were already using telehealth services, with urological practices experiencing significant uptake [14].

These early innovations in telehealth set the stage for the widespread adoption of telehealth technologies and integrated care models in urological care [15,16]. The development of video conferencing and real-time data transmission enabled health care providers to offer remote consultations, follow-ups, and monitoring. This approach addresses the problem of lengthy travel time for urology visits in rural areas. As a result, shifting towards telehealth facilitated remote consultations and follow-ups, seamlessly integrating other aspects of health care delivery and promoting a more coordinated and patient-centered approach [8].

**Figure 2.**
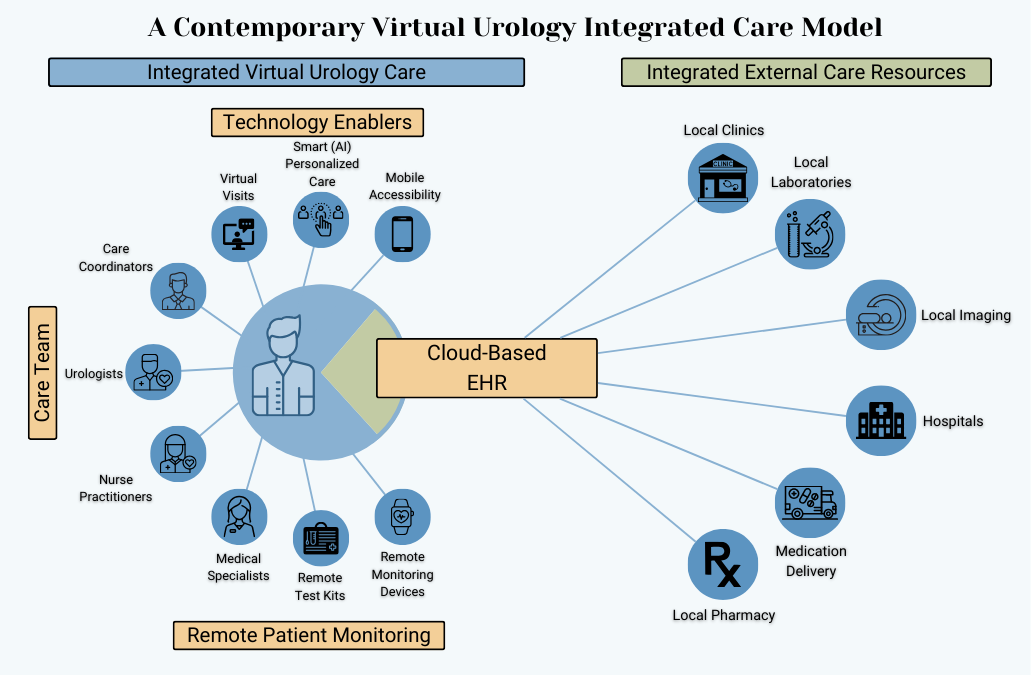
An overview of integrated care models. Patients are the focal point of technological innovations and collaboration between a network of caretakers [1,19]. AI: artificial intelligence; EHR: electronic health record.

As innovations in telehealth and technology contributed to an evolving health care landscape, the traditional fee-for-service model faced inconsistencies in care delivery and a lack of standardized care [17,18]. Since the late 20th century, Integrated care models that focus on improving quality and patient outcomes while reducing cost and waste are increasingly sought after [17]. Figure 2 demonstrates an overview of an integrated care model using telehealth systems in urology.

## Increasing Access and Patient Experience via Telehealth

Patients face access challenges to urologic care in both rural and urban settings. For starters, 61.4% of US counties lack a urologist, with less than 10% of primary urology practices residing outside metropolitan areas [20]. In rural Western states, the provider deficiency is larger than in Eastern states, due to sparsely populated areas. Therefore, most rural western counties lack timely access to a urologist within a 30-minute drive. Conversely, urban areas, despite having higher health care provider densities, face access limitations due to income inequality, lack of insurance, unaffordable co-pays, and uneven distribution of services. These issues particularly affect minority and lower socioeconomic status communities, resulting in poorer health outcomes [21].

Telemedicine has emerged as a transformative solution addressing access challenges in urology, particularly in rural areas where the shortage of urologists is pronounced [22-24]. By extending care to larger geographic regions with 24-hour direct-to-consumer virtual interactions, telehealth effectively bridges gaps in health care availability [22]. Studies underscore its role in overcoming socioeconomic disparities, with the recent inclusion of telehealth services by the Centers for Medicare & Medicaid Services (CMS) during public health emergencies. This significantly improves access for those unable to perform video visits [24].

Research indicates a strong patient preference for telemedicine. In 2016, a study evaluated the telehealth experiences of 150 veterans with hematuria. With their initial consultation via telephone, the mean satisfaction scores exceeded 9/10 for overall satisfaction, efficiency, convenience, friendliness, care quality, understandability, privacy, and professionalism. A total of 147 out of 150 (98%) patients in this study preferred telephone-based encounters to face-to-face clinic visits because they could avoid transportation-related issues and logistical clinic issues. In addition, 145.5 out of 150 (97%) of patients reported high-quality evaluation, underscoring telehealth’s ability to address geographic and socioeconomic barriers while maintaining high standards of care [25].

A barrier to telehealth adoption is that patients may not trust telehealth over in-person care. Studies indicate that patients belonging to minority racial groups tend to seek care in emergency departments over telemedicine [26]. There are trends of mistrust with digital platforms, as well as lack of trust with physicians over telehealth. The patient-clinician relationship isn’t strong already during in-person care for some minority groups, and their skepticism for health care can be exacerbated during telehealth appointments [26,27]. Distrust with clinicians negatively affects telehealth outcomes, but a higher degree of trust conversely leads to higher patient satisfaction with telehealth. Therefore, telehealth and general trust with clinicians need to be built up in parallel to have better patient outcomes [28].

The American Urological Association (AUA) 2023 census report demonstrates 62% of counties lack a urologist, and 25% of communities lacking a local urologist also lack access to broadband internet [20,22]. This digital divide is particularly pronounced among older adults, low-income populations, and ethnic and racial minorities who may not be as familiar or comfortable using technology for medical consultations [1,29]. Language barriers further complicate telehealth adoption; in communities where English is not the primary language, poor audio translation quality during video visits can lead to declined telehealth visits in favor of less effective phone consultations [30]. Less successful telehealth visits are also associated with a berth of different factors: patients of Hispanic or Latino race or ethnicity, patients insured by Medicare, Medicaid, or other noncommercial insurance, and patients of low socioeconomic status [31].

**Figure 3.**
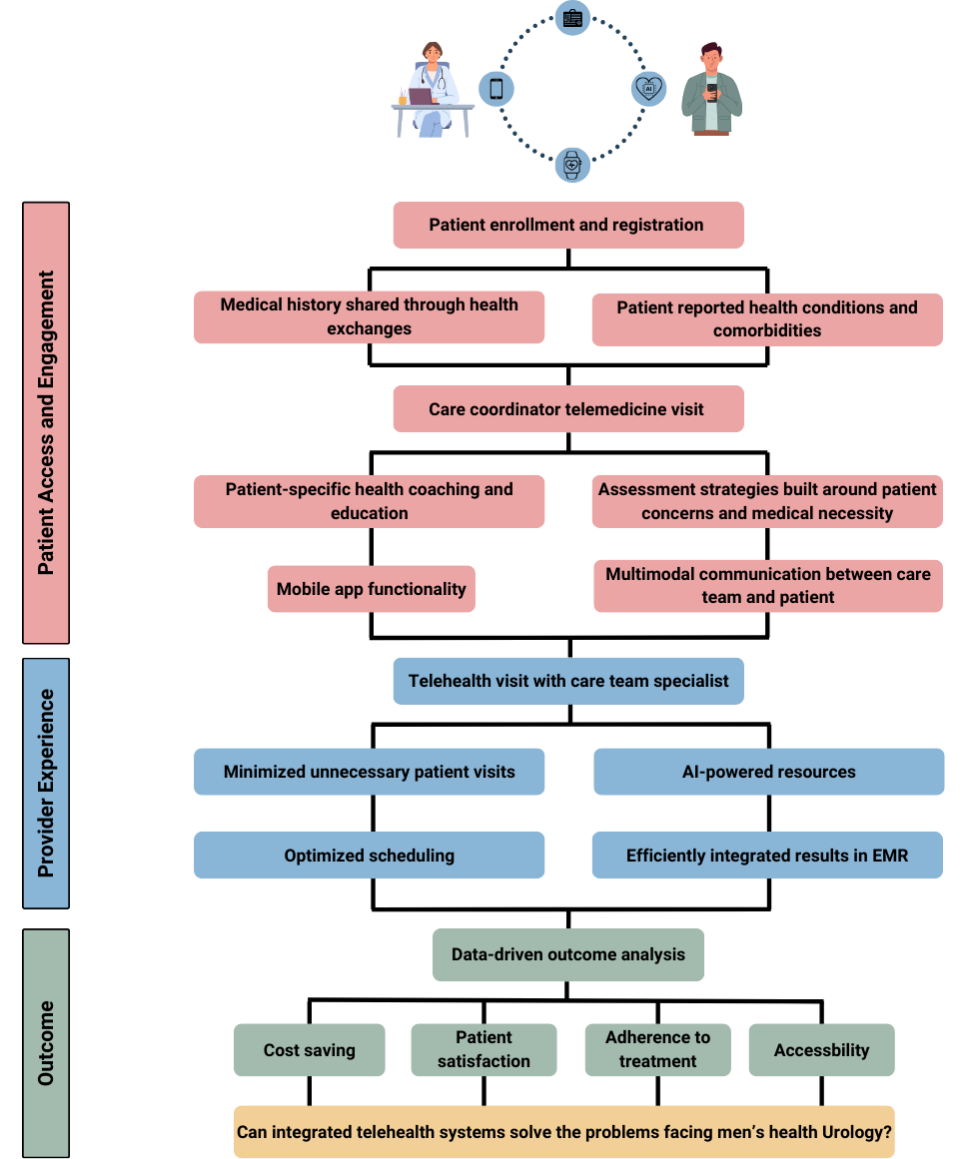
Depiction of the review process of components of telehealth systems affecting providers and patients. These factors are analyzed through outcome analysis and reviewed in the context of the quadruple aim of health care. AI: artificial intelligence; EMR: electronic medical record.

Addressing these patient-level barriers requires a multifaceted approach to enhance patients’ trust with telecommunications and clinicians, improve patients’ access and literacy with technology and the internet, and address the socioeconomic and ethnic divide that prevent patients from reaping the full benefits of telehealth [14,20,22,26-32]. Initiatives to provide affordable technology and improve digital literacy among older adults and vulnerable populations are crucial [14,29]. Integrating robust translation services into telehealth platforms can effectively address language barriers, facilitating better communication and care delivery [8,30]. Figure 3 shows the components of a telehealth system contributing to patient engagement and accessibility in the context of the quadruple aim of health care. Improving trust among minority patients can be facilitated by health care workers of minority backgrounds being messengers for health care innovations [32].

## Telehealth Provider Experience

The United States is facing a great shortage in the number of physicians. As a greater portion of the population gets older, telehealth solutions could provide a transformative approach to compensate for the surge. By 2030, it is estimated that 20% of the population will be aged 65 years and older, and this demographic shift is expected to create a 32% shortage in the number of urologists required to meet the demand of over 350 million US citizens [24]. In addition, the active urologist-to-population ratio has not kept pace with the growing population, indicating the potential of innovative solutions like telehealth to bridge this gap [33].

From 2016 to 2020, the overall burnout rate among urologists surged from 38.8% to 54%, while the overall physician burnout rate decreased to 41% by 2020 [34]. The 2023 AUA Census indicated that 71% of urologists have experienced burnout, with 85.6% of female urologists experiencing burnout. Furthermore, 33% of coping mechanisms by practicing urologists involved maladaptive behaviors such as drinking alcohol, eating junk food, and binge eating [20]. In addition, work-life balance remains a significant issue, with only 67.3% of urologists reporting sufficient time for family and personal life in the 2017 AUA Census. This percentage drops notably among female urologists, especially those aged 45 years and younger, where only 36.3% reported adequate work-life balance [34].

Telehealth has the potential to alleviate some of the pressures faced by urologists by reducing the need for in-person visits and allowing for more flexible scheduling. By enabling remote consultations, follow-ups, and even emergency interventions, telehealth can distribute the workload more evenly and provide support to areas with fewer health care resources [8]. During the COVID-19 pandemic, urology providers reported high satisfaction with telemedicine, appreciating its convenience and efficiency, indicating a preference to continue using telecommunication with patients [35]. Figure 3 demonstrates the components of telehealth systems contributing to improved provider experience in the context of the quadruple aim.

Furthermore, advancements in telemedicine technologies, such as AI and the Internet of Things (IoT), enhance the capabilities of health care providers. These technologies enable automatic note-taking through natural language processing, remote specialist consultations during emergency operations, and consolidated patient management through IoT platforms [8].

Despite innovations, health care providers face several barriers to telehealth adoption. One concern is extensive training on new telehealth technologies, which can be time-consuming and require high technological dexterity [14]. Providers are also concerned about the potential overload of patient data from home monitoring devices, which may be more than they can effectively manage. There is also resistance among clinical staff adjusting to the workflow changes that telehealth implementation necessitates, such as adapting to video visits and altered roles within the health care team [36,37].

Comprehensive training programs are essential to increase technological proficiency among health care professionals. These programs can ensure clinicians and staff are comfortable with telehealth technologies and can integrate them effectively into their workflows [14]. Streamlining telehealth data management systems to prevent information overload and support maintaining patient-provider relationships in a virtual environment can also mitigate provider resistance [36]. Addressing concerns about changes in workflow and roles through proper training and clear communication can further enhance telehealth adoption [14].

## Telehealth and Urology Cost-Savings

As life expectancy rises, an increasing number of older men are being diagnosed with cancer, particularly prostate cancer, which is the most commonly diagnosed malignancy among elderly males [38]. This brings forth the challenge of efficiently managing the long-term health and economic needs of an aging population [39].

Telehealth and integrated care models are well-suited to address these challenges by providing continuous, patient-centered care that adapts to older adults’ evolving health and economic needs [40]. For instance, stage 1 detection of prostate cancer through telehealth can save up to US $309,000 over four years compared to a stage 4 diagnosis [41]. Studies indicate that these models improve patient longevity and quality of life while managing the economic burden of chronic diseases [14,22,42].

An economic evaluation of telehealth appointments at a National Cancer Institute–Designated Comprehensive Cancer Center found that telehealth visits yielded considerable cost savings for patients. In a sample of 11,688 patients, 65 years and younger, who completed a total of 25,496 telehealth visits, patients saved on average US $147 to US $186 per visit. These savings were attributed to reduced travel costs or time lost from work [43].

A review found that telemedicine saved an average of US $149 to US $252 per patient visit in andrology, with patients showing a strong preference for videoconferencing over telephone visits, indicating that higher-quality telehealth interactions contribute to these savings [44]. Similarly, a study at the Moffitt Cancer Center analyzed over 25,000 telehealth visits, revealing substantial indirect cost savings. The study estimated savings from reduced lost productivity due to driving and visit time, averaging US $64.20 per telehealth visit and driving cost savings ranging from US $83.20 to US $122.00 per visit [43]. These savings do not account for additional time and cost savings for caregivers, who often provide transportation and lose productivity attending appointments [43,44].

Integrated care models also reduce health care costs. One approach identified rate-limiting steps and reduced redundancies, leading to a 69% decrease in per-patient clinic costs, from US $619 to US $194. In addition, the implementation of integrated care increased the number of patients seen per clinic day from 14 to 43 and improved family experience scores. These findings underscore the significant cost savings and operational efficiencies that integrated care models can achieve in health care settings [45].

Studies indicate that value-based care models in urology demonstrate significant potential for cost savings by enhancing efficiency and quality in urological practices [45-47]. Value-based programs in urology can reduce unnecessary procedures and hospital admissions, thus lowering overall health care expenditures [45]. Continuous quality improvement interventions, such as audited physician feedback and educational support, can streamline clinical practices, reduce redundancies, and promote cost-effective treatments like active surveillance for low-risk prostate cancer [46]. Furthermore, integrated care pathways and collaborative approaches in urology can reduce complications and associated costs [47]. A common consensus in urologic literature is that value-based care models prioritizing high-quality, coordinated care can drive down health care costs while maintaining or improving the standard of urological care.

## Telehealth and Urology Outcomes

Telehealth in urology has demonstrated significant benefits in patient satisfaction, health outcomes, and practicing preventive care, outweighing the benefits of in-person care in some situations [23,48]. A recent AUA study explored the standard of care for postoperative visits and clinically relevant visits in urology, comparing video visits to in-person visits. Within both groups, there were similar amounts of video and in-person visits, and neither visiting type resulted in any emergency room visits or hospitalizations within 30 days of their appointments [22].

Telemedicine also plays a critical role in enhancing preventive care, which is essential for managing long-term health outcomes. Telehealth offers a unique opportunity to enable early detection of conditions like cancer, reducing the need for radical treatments at later stages of this disease [42]. For instance, the 5-year survival rate for early-stage prostate cancer is nearly 100%, whereas prostate cancers detected at stage four have a 5-year survival rate of 28% [49,50]. Similarly, for colorectal cancer, the 5-year relative survival rate for stage 1 is about 92%, while stage IV metastatic colorectal cancer has a 5-year survival rate of only 12% [38]. These statistics highlight the importance of improving outcomes in men’s health urology through early detection and intervention [38,42,49,50]. Telehealth can facilitate this by making health care more accessible and convenient for patients [23,40].

Integrated care models can significantly improve patient outcomes in urology [40]. A 2017 study analyzed 72,411 prostate cancer patients across various market integration levels, finding that those in fully integrated markets had better outcomes, including higher rates of pretreatment counseling and appropriate imaging avoidance [51]. These patients were more likely to avoid unnecessary treatments when life expectancy was less than ten years and reduce multiple hospitalizations in the last 30 days of life. Another study reviewed integrated practice units for prostate cancer and lower urinary tract symptoms, noting increased active surveillance for low-risk prostate cancer, better adherence to guidelines for intermediate-risk disease, and greater use of androgen deprivation therapy for high-risk patients. They also observed fewer unnecessary cystoscopies and hospitalizations in multidisciplinary lower urinary tract symptoms (LUTS) clinics [45]. These findings underscore the efficacy of integrated care models in enhancing patient care quality and outcomes, although additional interventions like bundled payment models may further optimize health care value [45,51].

The transition to value-based care models is closely associated with enhanced patient outcomes in urology [45-47]. Such models have shown to improve patient health through better management and preventative services, leading to reduced hospitalizations and enhanced overall well-being [45]. In addition, value-based models demonstrate that continuous quality improvement initiatives, such as audited feedback and adherence to clinical guidelines, significantly improve clinical practices in urology. This is seen in the higher adoption rates of active surveillance in low-risk prostate cancer patients and better adherence to safety protocols like the prostate biopsy time-out, therefore reducing complications [46]. Golla et al [47] reinforces this by showing that coordinated care efforts and integrated health care delivery enhance the management of urological conditions, resulting in fewer complications and improved patient health outcomes. These findings collectively indicate that value-based care models not only optimize resource use but also significantly elevate the quality of patient care in urology, leading to better health outcomes [45-47].

## Future Directions

The findings of this viewpoint underscore the urgent need to reimagine men’s health urology through the lens of telehealth and integrated care. Traditional health care delivery models have long struggled to engage men—particularly in preventive and longitudinal care—resulting in delayed diagnoses, fragmented treatment, and suboptimal outcomes. Our analysis demonstrates that innovative telehealth integrated care models have the potential to directly confront these challenges. By improving access, enhancing patient engagement, reducing provider burnout, and lowering health care costs, these models offer a scalable solution to some of the most entrenched issues in men’s health care. Eventual implementation of these models require further research and action. We propose the following key recommendations to accelerate the adoption and impact of telehealth and integrated care in men’s health urology:

1. Establish standardized care protocols across telehealth platforms: to ensure consistent, evidence-based, and high-quality care, national guidelines specific to telehealth delivery in urology should be developed. These protocols will support clinical decision-making, reduce variability in care, and foster trust among providers and patients alike.
2. Integrate advanced digital health tools: the incorporation of wearable devices, mobile health apps, remote diagnostics, and AI-driven symptom monitoring can personalize care, improve early detection of urological conditions, and maintain patient engagement between visits. These tools transform passive care into an ongoing, interactive process.
3. Prioritize health equity in telehealth expansion: telehealth must not widen existing disparities. A targeted focus is needed to improve digital literacy, broadband access, and insurance coverage in underserved and rural communities. Partnerships with public health organizations and policymakers can ensure that these populations are not left behind.

## Conclusion

The implementation of telehealth and integrated care models in men’s health urology addresses the diverse and multifaceted challenges of this field. The complexities of managing common conditions such as prostate cancer, benign prostatic hyperplasia, and erectile dysfunction necessitate interdisciplinary solutions. A one-size-fits-all approach is insufficient for these varied health concerns. While the shift from traditional in-person care to telehealth and integrated models significantly enhances access and outcomes for a broader patient population, it still faces hurdles related to the integration of care across different platforms and providers. Telehealth breaks down barriers in men’s health by providing more flexible, accessible, and convenient care options, addressing issues of delayed engagement and limited access to urologists. Integrated care models, by breaking down the silos that traditionally impede communication and collaboration among health care providers, offer a solution to many of these challenges. However, the widespread adoption of these models will require overcoming significant structural, technological, and financial barriers. This viewpoint proposes changes that could reduce the significance of these barriers, providing health care leaders, policymakers, and urology providers a framework to tackle the challenges facing men’s health. Moving forward with these changes will require collaboration, a collective interest in innovation, and a willingness to challenge the traditional system that stands in the way of the future of men’s health urology.

## Abbreviations

AI: artificial intelligence
AUA: American Urological Association
CMS: Centers for Medicare & Medicaid Services
IoT: Internet of Things
LUTS: lower urinary tract symptoms
